# Topographic Relationships among Deep Optic Nerve Head Parameters in Patients with Primary Open-Angle Glaucoma

**DOI:** 10.3390/jcm11051320

**Published:** 2022-02-27

**Authors:** Do-Young Park, Hoon Noh, Changwon Kee, Jong-Chul Han

**Affiliations:** 1 Department of Ophthalmology, Yeungnam University Hospital, Yeungnam University College of Medicine, Daegu 42415, Korea; dypark@ynu.ac.kr; 2 Department of Ophthalmology, Samsung Medical Center, Sungkyunkwan University School of Medicine, Seoul 06351, Korea; appletea06@gmail.com (H.N.); ckee@skku.edu (C.K.); 3 Department of Medical Device, Management and Research, SAIHST, Sungkyunkwan University, Seoul 06355, Korea

**Keywords:** open-angle glaucoma, myopia, optic nerve head parameter, lamina cribrosa, optical coherence tomography

## Abstract

Purpose: To investigate the topographic relationships among the deep optic nerve head (ONH) parameters representing myopic axial elongation or changes in the lamina cribrosa (LC) in patients with primary open-angle glaucoma (POAG). Methods: Among patients with POAG who visited the clinic between January 2015 and March 2017, the following deep ONH parameters were measured using spectral-domain optical coherence tomography (SD-OCT): externally oblique border tissue (EOBT) length, ONH tilt angle, optic canal (OC) obliqueness, and anterior LC insertion depth (ALID). In addition, the angular locations of the maximal value of each parameter were measured. We analyzed the correlations between the parameters, correlations with axial length (AL), and the spatial correspondence with glaucomatous ONH damage. Results: A total of 100 eyes with POAG were included in the analysis. The EOBT length, ONH tilt angle, and OC obliqueness were correlated with each other and with AL, whereas ALID showed less correlation with the other parameters and AL. The angular location where the three AL-related parameters had maximum values was also correlated with the predominant region of the glaucomatous ONH damage, while the angular location of the deepest ALID showed less correlation. Conclusions: Among the deep ONH parameters, the AL-related parameters EOBT length, ONH tilt angle, and OC obliqueness showed strong spatial correspondence with glaucomatous ONH damage, whereas the LC-related parameter ALID was less correlated with both AL and the region with glaucomatous ONH damage. Further studies are needed to determine how these differences affect glaucomatous ONH change.

## 1. Introduction

Deep optic nerve head (ONH) structures such as the parapapillary sclera, scleral canal wall, and lamina cribrosa are known to be closely related to the development of glaucomatous optic disc damage [1,2,3]. Although intraocular pressure (IOP) plays the most important role in glaucomatous ONH injury, the location of optic disc damage cannot be predicted by IOP. On the other hand, the deep ONH structures and their locational properties are deeply associated with the site of the glaucomatous ONH damage [4,5,6,7]. Visualization of deep ONH structures such as Bruch’s membrane (BM) openings or border tissue of Elschnig using optical coherence tomography (OCT) has made it possible to evaluate the association between deep ONH structures and glaucomatous damage by objectively measuring the ONH parameters [8,9,10,11].

In our prior studies, we measured deep ONH parameters including externally oblique border tissue (EOBT) length, ONH tilt angle, and optic canal (OC) obliqueness and found that these characteristics were associated with the presence of glaucoma and the location of glaucomatous damage in eyes with myopic normal tension glaucoma [6,7]. In addition to these parameters, anterior lamina cribrosa insertion depth (ALID) has recently been demonstrated to represent the posterior migration of the laminar insertion and is displaced more posteriorly in eyes with POAG than in healthy eyes [12,13,14,15,16]. However, how the LC-related parameter ALID is associated with other deep ONH parameters such as EOBT length, ONH tilt angle, and OC obliqueness, or myopic axial elongation, remains unclear.

Thus, we measured four deep ONH parameters in this study: EOBT length, ONH tilt angle, OC obliqueness, and ALID using SD-OCT in patients with POAG. Then, we investigated correlations between the parameters, the correlation with axial length (AL), and spatial correspondence with glaucomatous ONH damage.

## 2. Methods

### 2.1. Participants

For this cross-sectional observational study, patients with OAG (with myopia less than −0.5 diopters (D)) who visited Samsung Medical Center (Seoul, Korea) for their first ophthalmic examination between January 2015 and March 2017 were reviewed, and patients who met the inclusion and exclusion criteria were included. This study was approved by the Institutional Review Board (IRB)/Ethics Committee of Samsung Medical Center and followed the tenets of the Declaration of Helsinki.

The inclusion criteria were as follows: (1) patients diagnosed with OAG at least in one eye after a comprehensive ophthalmic examination; (2) patients with myopia less than −0.5 D in eyes with OAG; and (3) patients with ONH with visible EOBT on OCT examination in eyes with OAG. Patients or eyes satisfying the following criteria were not included: (1) eyes with media opacities, such as a corneal or vitreous opacity or moderate to severe cataract; (2) patients who had systemic or ocular diseases that could affect VF test results; (3) eyes with a high degree of myopia with AL > 28 mm accompanied by myopic degeneration or retinal schisis around the ONH; and (4) eyes with a VF MD of −12 dB or less, for which the glaucomatous VF pattern would be difficult to determine. If the patient had OAG in both eyes, only the eye with less-severe MD was included in the analysis.

Each participant underwent a comprehensive ophthalmic examination, including slit-lamp biomicroscopy, Goldmann applanation tonometry (GAT), manifest refraction, gonioscopic examination, dilated stereoscopic examination of the ONH, color and red-free fundus photography (TRC-50DX; Topcon Medical System, Inc., Oakland, NJ, USA), automated perimetry using a central 30–2 Humphrey Field Analyzer (HFA, model 640; Humphrey Instruments, Inc., San Leandro, CA, USA) with the Swedish interactive threshold algorithm standard, AL measurement (IOL Master; Carl Zeiss Meditec, Jena, Germany), ultrasound pachymetry (Tomey SP-3000; Tomey Ltd., Nagoya, Japan), and SD-OCT examination (Heidelberg Engineering, Heidelberg, Germany). The extent of the VF defect was measured using the mean deviation (MD), pattern standard deviation (PSD), and visual field index (VFI). Reliable VF analysis was defined as a false-negative rate < 15%, a false-positive rate < 15%, and a fixation loss of <20%. IOPs were measured at the first and second visits without IOP-lowering medications. Average IOP values were used in the analysis.

OAG was diagnosed based on the following criteria: (1) the presence of glaucomatous optic disc changes, such as increased cupping (vertical cup-to-disc ratio > 0.7), diffuse or focal neuroretinal rim thinning, disc hemorrhage, or RNFL defects; (2) an open angle on gonioscopic examination with no identifiable causes of secondary glaucoma; and (3) glaucomatous VF defects positive by more than one reliable test for at least two of the following criteria: (1) a cluster of three points with a probability less than 5% on the pattern deviation map in at least one hemifield, including at least one point with a probability less than 1% or a cluster of two points with a probability less than 1%; (2) a glaucoma hemifield test result outside the normal limits; or (3) a PSD of 95% outside the normal limits.

### 2.2. Imaging of Optic Nerve Head Using SD-OCT

For imaging of the deep structure of the ONH, spectral-domain OCT (SD-OCT; Heidelberg Engineering) with the enhanced depth-imaging (EDI) mode was used. Details of the methods were given previously [7]. Briefly, 48 radial B-scan images (interval of 3.75°) centered on the optic disc were acquired using the EDI mode. Each scan included an average of 20 OCT frames. Magnification errors were corrected using a formula provided by the manufacturer based on results of autorefraction keratometry and focus setting during image acquisition. To measure the parameters of the deep ONH, every other section among the 48 scans (24 radial EDI scans in total) was selected and the scaling was adjusted to 1:1 μm in the software. If the scan section contained a poor-quality OCT image that did not provide interpretable information regarding the BMO or border tissue due to the presence of prelaminar tissue or overlying vessels, the next image was used. If more than three of the twenty-four radial scans were unrecognizable, the eye was excluded from the analysis.

The presence of EOBT on OCT images was assessed by two investigators (HN and JCH) in a masked fashion. Disagreement between the investigators was resolved by a third adjudicator (CK).

### 2.3. Measurement of the Extent and Angular Location of Deep ONH Parameters Using SD-OCT

In a previous study, we defined several parameters representing deep ONH structures on OCT images, such as EOBT length, ONH tilt angle, and optic canal (OC) obliqueness [7]. As previously described, EOBT length was defined as the length between the two end points of the EOBT tissue and ONH tilt angle was defined as the angle between the BMO plane and the optic canal plane. The BMO plane was defined as the line connecting the two BMOs, nasal and temporal. The optic canal plane was defined as the line connecting the nasal BMO and the innermost margin of the EOBT. OC obliqueness was defined as the angle formed by a vertical line and the EOBT [7]. Anterior lamina cribrosa insertion (ALI) was also defined as previously described as the intersection of the scleral canal wall and the anterior surface of the lamina cribrosa in each of the 24 radial scans [12]. ALI depth (ALID) was defined as the distance from the anterior scleral canal opening (ASCO) to the ALI. The maximal value of each parameter among all scanned sections measured was defined as the maximum deep ONH parameter.

**Figure 1:**
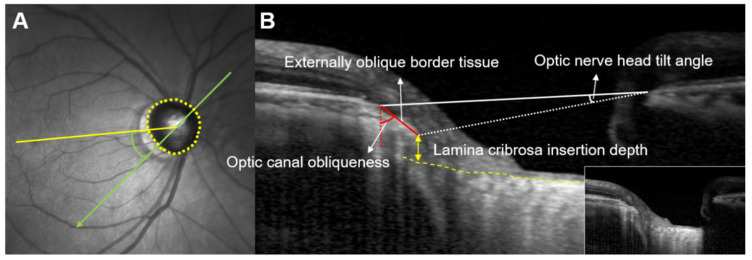
Measurement of the extent and location of the deep ONH parameters EOBT length, ONH tilt angle, OC obliqueness, and ALID. (**A**) BMOs (yellow dots) were marked and the FoBMO axis (yellow line), which is the line connecting the center of the BMO and fovea, was used as the reference line. The locations of maximum deep ONH parameters were measured as angles (green) from the FoBMO axis. When the directions were located below the FoBMO axis, they were assigned positive values. (**B**) Methods for measuring EOBT length, ONH tilt angle, OC obliqueness, and ALID are displayed.

The angular location of the maximum deep ONH parameters was measured using the infrared (IR) scanning laser ophthalmoscopy (SLO) image provided by SD-OCT. The line connecting the center of the BMO and fovea was defined as the fovea–BMO (FoBMO) axis. In case the IR photo did not contain the fovea, we set the position of the fovea after aligning the IR and red-free photos using Photoshop CS5 (Adobe System, San Jose, CA, USA). The angular location was defined as the angle between the location of each maximum deep ONH parameter and the FoBMO axis. If the angular location of each parameter was below the FoBMO axis, the location was assigned a positive value. Otherwise, the location of each parameter was assigned a negative value. A schematic diagram of these parameters is provided in the authors’ previous work [6,7] (Figure 1).

The extent and angular location of all parameters described above were assessed by two investigators (HN and JCH), and the average values of the two investigators were used in the final analysis.

### 2.4. Determination of Dominant VF Defect Locations

We divided the VF defect patterns based on the dominant VF defect location (superior vs. inferior dominant). To determine the location of the dominant VF defect, we calculated the average values in pattern deviation plots at the superior and inferior hemifield, respectively (26 points in each hemifield). When one hemifield had a greater absolute value than the other, we regarded it as a dominant VF defect location.

### 2.5. Statistical Analysis

The intraobserver (two consecutive measurements by HN) and interobserver (measured by HN and JCH) reproducibility of measurements of the OCT parameters were assessed by calculating the intraclass correlation coefficients (ICCs). SD-OCT images of 20 randomly selected patients were used for this analysis. To compare the mean values of continuous variables between the two subgroups, the Mann–Whitney *U* test was used. A Chi-square test or Fisher’s exact test was performed for comparison of categorical variables. Correlations between the parameters and AL and correlations among the parameters were evaluated by Pearson’s correlation analysis, and the correlation coefficient (R) was calculated. The Brown–Forsythe test was performed to assess the equality of distribution of the parameters. An AL of 25 mm was arbitrarily set as a cutoff for comparison between the two subgroups according to AL. A logistic regression analysis was performed to confirm the factors associated with the location of the dominant VF defect. A *p*-value < 0.05 was considered statistically significant. Statistical analyses were performed using IBM SPSS software version 24.0 (IBM Corp., Armonk, NY, USA) and R statistical package version 3.5.3 (R Foundation for Statistical Computing, Vienna, Austria).

## 3. Results

A total of 139 eyes were analyzed from 139 enrolled patients. Among these eyes, 21 (15.1%) were excluded because of the poor quality of OCT images that did not allow for clear visualization of deep ONH structures such as the BMO or EOBT. Eight eyes with unreliable VF tests, six eyes of advanced glaucoma with VF MD < −12 dB, and four highly myopic eyes with AL > 28 were also excluded from the analysis. In total, 100 eyes from 100 patients were included in the analysis. Intraobserver and interobserver ICCs showed good agreement for the assessment of the extent and the angular location of all four parameters (Table S1). Baseline characteristics of the subjects are described in Table 1.

### 3.1. Spatial Correspondence between Deep ONH Structures and VF Defects

When the eyes were divided into two groups according to the location of the predominant VF defect, there were 68 (68.0%) eyes in the superior dominant group and 32 (32.0%) eyes in the inferior dominant group. Baseline characteristics, such as age, IOP, AL, CCT, and MD, did not show significant differences between the two groups. The maximum deep ONH parameters, such as EOBT length, ONH tilt angle, OC obliqueness, and ALID, did not differ between the two groups in terms of their extent. However, all the maximum deep ONH parameters except for ALID were significantly inferiorly located in the superior dominant VF group compared with the inferior dominant VF defect group (*p* = 0.008 for maximum EOBT length; *p* = 0.008 for maximum ONH tilt angle; *p* = 0.001 for maximum OC obliqueness; *p* = 0.150 for maximum ALID) (Table 1). A logistic regression analysis also showed that the angular locations of all the deep ONH parameters except for ALID were significantly associated with the inferiorly located dominant VF defect (Table S2). When the angular locations were divided by binary values of positive and negative directions relative to the FoBMO axis, the binary values of the locations of all maximum deep ONH parameters were significantly associated with the dominant location of the VF defect (*p* = 0.026 for the maximum EOBT length location; *p* = 0.035 for the maximum ONH tilt angle location; *p* = 0.001 for the maximum OC obliqueness location; *p* = 0.028 for the maximum ALID location) (Table 1).

### 3.2. Extent and Location of Deep ONH Parameters According to AL

When we analyzed the correlation between the deep ONH parameters, significant correlations were observed between all parameters except ALID in both aspects of the extent and angular location (Table 2).

**Figure 2:**
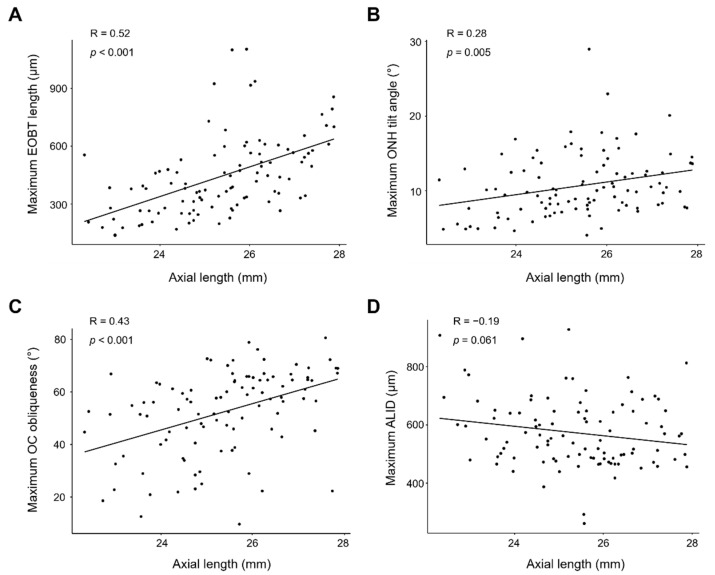
Pearson’s correlation plots showing correlations between AL and maximal EOBT length (**A**), maximal ONH tilt angle (**B**), maximal OC obliqueness (**C**), and ALID (**D**). All parameters except ALID showed significant correlations with AL.

Next, we analyzed the correlation between the AL and the extent of deep ONH parameters. The extent of all deep ONH parameters except ALID were significantly positively correlated with AL (R = 0.52, *p* < 0.001 for the maximum EOBT length; R = 0.28, *p* = 0.005 for the maximum ONH tilt angle; R = 0.43, *p* < 0.001 for the maximum OC oblique-ness; R = −0.19, *p* = 0.061 for the maximum ALID) (Figure 2).

**Figure 3:**
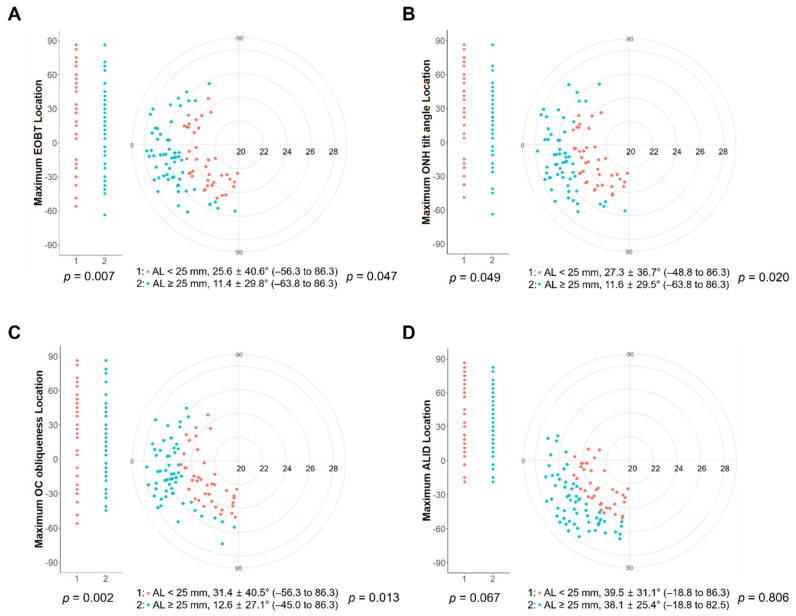
Frequency distribution of the location of each deep ONH parameter with the maximal value (maximal EOBT length (**A**), maximal ONH tilt angle (**B**), maximal OC obliqueness (**C**), and ALID (**D**)) according to the meridian clock. The eyes were divided into two groups with a cutoff of 25 mm. In eyes with AL ≥ 25 mm, all the maximum deep ONH parameters except ALID were significantly located temporally compared with the eyes with AL < 25 mm. All of the deep ONH parameters with their maximal values were placed in the inferotemporal direction. In eyes with AL ≥ 25 mm, all the parameters except ALID showed narrower distributions than in eyes with AL < 25 mm.

The angular locations of the parameters are shown in Figure 3. The angular locations of the deep ONH parameters were mainly positioned in the inferotemporal region of the ONH. When the AL was divided into two groups (AL ≥ 25 mm and AL < 25 mm), in eyes with AL ≥ 25 mm, the maximum EOBT location, maximum ONH tilt angle location, and maximum OC obliqueness location were located more superiorly (temporally) than in eyes with AL < 25 mm, while the maximum ALID location was not significantly different between the two groups (*p* = 0.047 for the maximum EOBT length; *p* = 0.020 for the maximum ONH tilt angle; *p* = 0.013 for the maximum OC obliqueness; *p* = 0.806 for the maximum ALID). In addition, eyes with AL ≥ 25 mm showed significantly narrower distributions in the maximum EOBT location, maximum ONH tilt angle location, and maximum OC obliqueness location than those with AL < 25 mm, while the maximum ALID did not (*p* = 0.007 for the maximum EOBT length; *p* = 0.049 for the maximum ONH tilt angle; *p* = 0.002 for the maximum OC obliqueness; *p* = 0.067 for the maximum ALID).

### 3.3. Relationship between RNFL Thickness and Location of Deep ONH Parameters

We divided the deep ONH parameters into ‘superior’ and ‘inferior’ groups according to the position of their maximum values. When we compared the RNFL thickness at the superior, temporal, inferior, and nasal positions between the two groups of each deep ONH parameter, the inferior RNFL thickness was significantly lower in inferiorly located groups with the parameters of EOBT location, ONH tilt location, and OC obliqueness location compared with superiorly located groups. On the other hand, there was no significant difference in the inferior RNFL thickness according to the location of the maximum ALID (Table 3).

## 4. Discussion

In this study, we analyzed the characteristics of four deep ONH parameters measured by SD-OCT in patients with POAG in terms of correlations between the parameters, correlations with AL, and spatial correspondence with the glaucomatous ONH damage. As a result, we found that EOBT length, ONH tilt angle, and OC obliqueness, which are known to be regionally correlated with PPA, were correlated with each other and with AL, whereas ALID showed less correlation with other parameters and AL. In addition, the location where these three inter-related parameters had maximum values corresponded well to the predominant region of the glaucomatous ONH damage, while the location where ALI was the deepest corresponded less. These results indicate that each of the parameters representing the features of deep ONH may have varying degrees of association with myopia or the location of the glaucomatous damage, which should be taken into account when we interpret the myopic or glaucomatous ONH change.

In this study, four parameters were investigated as parameters characterizing the deep ONH: EOBT length, ONH tilt angle, OC obliqueness, and ALID. Of these, as previously reported, the extent of EOBT length, ONH tilt angle, and OC obliqueness, which were associated with the presence of PPA, were strongly correlated with the AL [7]. The location of their maximum values was positioned temporally as the AL elongated. In addition to these three parameters, we analyzed ALID in this study. ALI is the part of the wall of the scleral canal in contact with the anterior surface of the LC, and its depth (ALID) can represent how much the LC is deformed posteriorly [17]. ALID has been reported to be located deeply in superior and inferior parts of the optic disc of healthy controls, and another study showed that ONHs of patients with POAG had a deeper ALID than those of healthy controls [12,13,14]. Therefore, ALID has been considered to be a factor related to the development of glaucomatous ONH change.

The interesting finding of this study was that, compared with the other three parameters, ALID had less correlation with the other parameters and with AL. We interpreted the reason for such a difference between the parameters as follows. When the AL is elongated and glaucomatous ONH changes occur at the same time, the ONH undergoes both passive changes and active remodeling. Among the four parameters we analyzed in this study, EOBT length, ONH tilt angle, and OC obliqueness are thought to be parameters representing the passive changes that occur as the AL increases based on findings that they show a strong correlation with AL [7]. On the other hand, ALID is a factor related to the active remodeling of the ONH occurring at the level of the LC [12,17], and it may not be directly related to the changes in AL. In other words, unlike the other three parameters, ALID can change even after the myopic AL elongation process stops.

In the analysis of the relationship between the positional characteristics of four ONH parameters and the predominant region of the glaucomatous ONH damage, contrary to our expectations, the AL-related factors EOBT length, ONH tilt angle, and OC obliqueness corresponded more to the location of glaucomatous ONH damage compared with the LC-related factor ALID. If the deepening of ALI is a phenomenon that reflects the glaucomatous change in the LC, ALID would seem to correlate better with the location of glaucomatous damage, but this was not the case in this study. This may be related to the fact that most patients (95%) included in this study had early stage normal tension glaucoma with a mild VF defect (MD −3.9 dB). Disc cupping due to elevated IOP may deepen ALI as the LC is excavated posteriorly, but disc cupping in eyes with NTG more involves focal prelaminar and neuroretinal rim thinning than lamina deformation, especially in early stage glaucoma [12,18,19]. In addition, even if the LC depth increases as glaucoma progresses, this phenomenon may occur in a diffuse manner rather than a localized manner, and the ALID will have a less localized value to correlate with glaucomatous damage. We think that parameters such as BMO-MRW representing the prelamina or neuroretinal rim can be correlated better in terms of the location of the glaucomatous ONH damage in eyes with NTG [20]. Analyzing more patients separately from eyes with NTG and POAG in the future will help to determine whether the topographical relationship between ALID and glaucomatous ONH damage is affected by IOP or the severity of glaucoma.

This study has several limitations. First, this study was a cross-sectional study, so it is not possible to establish causal relationships. In other words, the issues of whether parameters related to AL affect the location of the glaucomatous damage and whether changes in the ONH parameters and glaucomatous damage occur together due to other unknown factors remain unresolved. Similarly, the ALID can be deepened as the LC excavates backward as glaucoma progresses; however, on the other hand, an ONH with a deeper ALID may be more prone to glaucoma. Second, although the positions of each ONH parameter were measured precisely by angle, the location of glaucomatous damage in the ONH was inferred by comparing the superior and inferior regions of the VFD or RNFL thickness. If the thinnest location of the BMO-MRW is measured by angle in the future, it will provide a more detailed correlation analysis between the ONH parameters and the location of ONH damage. Finally, this study included a single disease group with a limited sample size. Further studies are needed to examine the differences between the parameters analyzed in this study, including the longitudinal changes, with a larger number of patients and healthy controls.

In conclusion, we found that among the deep ONH parameters, EOBT length, ONH tilt angle, and OC obliqueness were strongly correlated with AL and the location of the glaucomatous ONH damage whereas the LC-related parameter ALID was correlated with neither AL nor the region with glaucomatous ONH damage in eyes of patients with POAG. These findings suggest that AL-related ONH parameters and LC-related ONH parameters may have different influences on the localization of glaucomatous ONH damage. Further studies are needed to determine how these differences affect the development of glaucomatous ONH damage.

## Figures and Tables

**Table 1:** Baseline characteristics and deep optic nerve head parameters of the patients depending on the dominant VF involvement.

	Total (*n* = 100)	Superior Dominant VF Defect (*n* = 68)	Inferior Dominant VF Defect (*n* = 32)	*p* Value
Age, years	50.7 ± 11.7	49.4 ± 12.2	53.5 ± 10.4	0.102 *
IOP, mmHg	16.8 ± 3.1	16.8 ± 2.6	16.7 ± 3.9	0.921 *
AL, mm	25.4 ± 1.4	25.4 ± 1.5	25.5 ± 1.2	0.699 *
CCT, µm	538.8 ± 35.0	541.2 ± 36.4	533.7 ± 31.9	0.319 *
MD, dB	−3.9 ± 2.9	−4.1 ± 2.8	−3.4 ± 3.0	0.262 *
Deep ONH parameters				
Extent				
Maximum EOBT length, µm	445.0 ± 203.2	456.4 ± 213.2	420.8 ± 181.0	0.417 *
Maximum ONH tilt angle, °	10.6 ± 4.2	10.6 ± 4.2	10.6 ± 4.3	0.962 *
Maximum OC obliqueness, °	52.4 ± 16.0	51.2 ± 16.3	54.7 ± 15.4	0.309 *
Maximum ALID, µm	573.0 ± 119.1	579.4 ± 119.1	559.5 ± 119.8	0.440 *
Angular location, °				
Maximum EOBT location	16.9 ± 34.9	23.2 ± 34.0	3.5 ± 33.5	**0.008** *
Maximum ONH tilt angle location	17.7 ± 33.2	23.7 ± 33.3	5.0 ± 29.8	**0.008** *
Maximum OC obliqueness location	20.0 ± 34.0	27.4 ± 31.0	4.2 ± 35.3	**0.001** *
Maximum ALID location	38.6 ± 27.6	41.4 ± 25.9	32.8 ± 30.5	0.150 *
Inferior dominant location, *n* (%)				
Maximum EOBT location	71 (71.0)	53 (77.9)	18 (56.3)	**0.026** ^†^
Maximum ONH tilt angle location	73 (73.0)	54 (79.4)	19 (59.4)	**0.035** ^†^
Maximum OC obliqueness location	74 (74.0)	57 (83.8)	17 (53.1)	**0.001** ^†^
Maximum ALID location	91 (91.0)	65 (95.6)	26 (81.3)	**0.028** ^†^

VF, visual field; IOP, intraocular pressure; AL, axial length; CCT, central corneal thickness; MD, mean deviation; ONH, optic nerve head; EOBT, externally oblique border tissue; OC, optic canal; ALID, anterior lamina cribrosa insertion depth; °: degree. Values are mean ± SD or frequency (%); *p* values < 0.05 are shown in bold; * Independent *t*-test; † χ^2^ test or Fisher’s exact test for comparison between superior dominant VF defects and inferior dominant VF defects.

**Table 2:** Correlations among the extent and location of the deep ONH parameters.

**Parameters, Extent**	**Maximum** **ONH Tilt Angle**	**Maximum** **OC Obliqueness**	**Maximum ALID**
Maximum EOBT			
Pearson’s coefficient	0.707	0.577	−0.050
*p* value *	**<0.001**	**<0.001**	0.619
Maximum ONH tilt angle			
Pearson’s coefficient		0.255	0.149
*p* value *		**0.011**	0.140
Maximum OC Obliqueness			
Pearson’s coefficient			−0.172
*p* value *			0.087
**Parameters, Location**	**Maximum ONH Tilt Angle Location**	**Maximum OC Obliqueness Location**	**Maximum ALID Location**
Maximum EOBT location			
Pearson’s coefficient	0.883	0.819	0.290
*p* value *	**<0.001**	**<0.001**	**0.003**
Maximum ONH tilt angle location			
Pearson’s coefficient		0.803	0.159
*p* value *		**<0.001**	0.114
Maximum OC Obliqueness location			
Pearson’s coefficient			0.243
*p* value *			**0.015**

ONH, optic nerve head; EOBT, externally oblique border tissue; OC, optic canal; ALID, anterior lamina cribrosa insertion depth. *p* values < 0.05 are shown in bold; * Pearson’s correlation.

**Table 3:** Comparisons of RNFL thickness according to the location of the deep ONH parameters.

	**Inferior Maximum EOBT Location, *n* = 71**	**Superior Maximum EOBT Location, *n* = 29**	***p* Value**
RNFL thickness, µm			
Superior	93.5 ± 16.8 (58 to 138)	95.8 ± 18.8 (64 to 145)	0.555 *
Temporal	64.1 ± 12.5 (37 to 97)	64.9 ± 11.0 (41 to 81)	0.761 *
Inferior	77.8 ± 15.0 (47 to 117)	92.7 ± 17.2 (65 to 129)	**<0.001** *
Nasal	63.1 ± 8.9 (43 to 87)	64.4 ± 9.2 (49 to 84)	0.508 *
	**Inferior Maximum ONH Tilt Location, *n* = 73**	**Superior Maximum ONH Tilt Location, *n* = 27**	***p* Value**
RNFL thickness, µm			
Superior	94.6 ± 16.9 (58 to 138)	93.1 ± 18.8 (64 to 145)	0.720 *
Temporal	64.5 ± 12.2 (37 to 97)	63.9 ± 11.8 (41 to 89)	0.829 *
Inferior	79.4 ± 15.7 (47 to 129)	89.5 ± 18.5 (55 to 125)	**0.008** *
Nasal	62.7 ± 8.8 (43 to 87)	65.5 ± 9.3 (49 to 84)	0.168 *
	**Inferior Maximum OC Obliqueness Location, *n* = 74**	**Superior Maximum OC Obliqueness Location, *n* = 26**	***p* Value**
RNFL thickness, µm			
Superior	94.3 ± 17.1 (58 to 145)	93.8 ± 18.4 (64 to 129)	0.910 *
Temporal	64.2 ± 12.5 (37 to 97)	64.6 ± 10.7 (41 to 81)	0.885 *
Inferior	79.0 ± 14.5 (47 to 117)	90.1 ± 19.6 (55 to 129)	**0.002** *
Nasal	63.7 ± 9.2 (43 to 87)	62.7 ± 8.5 (49 to 83)	0.623 *
	**Inferior Maximum ALID location, *n* = 91**	**Superior Maximum ALID location, *n* = 9**	***p* Value**
RNFL thickness, µm			
Superior	94.7 ± 17.7 (58 to 145)	89.2 ± 13.0 (71 to 115)	0.323 *
Temporal	64.3 ± 12.3 (37 to 97)	64.9 ± 9.0 (53 to 78)	0.763 *
Inferior	81.5 ± 17.3 (47 to 129)	88.1 ± 14.1 (73 to 120)	0.250 *
Nasal	63.3 ± 9.1 (43 to 87)	65.0 ± 7.0 (57 to 76)	0.531 *

ONH, optic nerve head; EOBT, externally oblique border tissue; OC, optic canal; ALID, anterior lamina cribrosa insertion depth; RNFL, retinal nerve fiber layer. Values are mean ± SD (range); *p* values < 0.05 are shown in bold; * Independent *t*-test or Mann–Whitney *U* test.

## Data Availability

The data presented in this study are available from the authors upon reasonable request. The data are not publicly available due to privacy and ethical issue.

## Supplementary Materials

The following supporting information can be downloaded at: https://www.mdpi.com/article/10.3390/jcm11051320/s1, Table S1: Intra- and inter-observer reproducibility in measurement of parameters; Table S2: Factors associated with an inferiorly located dominant VF defect.
