# Supplementary material for: Topographic Relationships among Deep Optic Nerve Head Parameters in Patients with Primary Open-Angle Glaucoma
Source: J Clin Med. 2022 Feb 27;11(5):1320. doi: 10.3390/jcm11051320 (PMC8910857; doi:10.3390/jcm11051320)
Supplement: Supplementary file 1 [file jcm-11-01320-s001.zip › jcm-1556536-supplementary.pdf]

Table S1. Intra- and inter-observer reproducibility in measurement of parameters

Parameters	Intraobserver ICC (95% CI)	Interobserver ICC (95% CI)
Extent		
Maximal EOBT length, μm	0.979 (0.955–0.995)	0.968 (0.926–0.992)
Maximal ONH tilt angle, °	0.975 (0.932–0.985)	0.947 (0.895–0.982)
Maximal OC obliqueness, °	0.981 (0.938–0.992)	0.951 (0.901–0.984)
Maximal ALID, μm	0.972 (0.967–0.989)	0.962 (0.924–0.991)
Angular location		
Maximal EOBT location, °	0.948 (0.903–0.989)	0.922 (0.835–0.972)
Maximal ONH tilt location, °	0.962 (0.927–0.992)	0.936 (0.882–0.988)
Maximal OC obliqueness location, °	0.945 (0.911–0.972)	0.931 (0.854–0.984)
Maximal ALID location, °	0.951 (0.926–0.988)	0.926 (0.893–0.956)

ONH: optic nerve head; EOBT: externally oblique border tissue; OC: optic canal; ALID: anterior lamina cribrosa insertion depth; ° degree.

Table S2. Factors associated with an inferiorly located dominant VF defect

	Univariate		Multivariate					
			Model 1		Model 2		Model 3	
Variables	**OR (95% CI)**	***p* value**	**OR (95% CI)**	***p* value**	**OR (95% CI)**	***p* value**	**OR (95%CI)**	***p* value**
Age, years	1.03 (0.99,1.07)	0.104	1.05 (1,1.11)	0.055	1.05 (1,1.11)	0.072	1.04 (0.99,1.1)	0.105
IOP, mmHg	0.99 (0.86,1.14)	0.908	1.02 (0.88,1.2)	0.760	1 (0.86,1.17)	0.952	1.01 (0.86,1.18)	0.934
AL, mm	1.06 (0.78,1.45)	0.696	1.33 (0.82,2.16)	0.255	1.35 (0.83,2.2)	0.230	1.26 (0.77,2.06)	0.354
CCT, μm	0.99 (0.98,1.01)	0.317	0.99 (0.98,1)	0.184	0.99 (0.98,1)	0.189	0.99 (0.98,1.01)	0.285
MD, dB	1.09 (0.94,1.28)	0.262	1.06 (0.89,1.27)	0.489	1.08 (0.91,1.3)	0.377	1.07 (0.89,1.28)	0.474
Maximum EOBT length, μm	1 (1,1)	0.414	0.99 (0.99,1)	0.069	0.99 (0.99,1)	**0.046**	0.99 (0.99,1)	0.056
Maximum ONH tilt angle, °	1 (0.9,1.1)	0.962	1.07 (0.89,1.3)	0.466	1.09 (0.9,1.32)	0.354	1.1 (0.9,1.33)	0.359
Maximum OC obliqueness, °	1.01 (0.99,1.04)	0.307	1.04 (0.99,1.09)	0.100	1.04 (1,1.09)	0.074	1.04 (0.99,1.09)	0.113
Maximum ALID, μm	1 (0.99,1)	0.437	1 (1,1)	0.869	1 (1,1)	0.885	1 (1,1)	0.931
Maximum EOBT angular location, °	0.98 (0.97,1)	**0.010**	0.98 (0.96,1)	**0.013**				
Maximum ONH tilt angle angular location, °	0.98 (0.97,1)	**0.011**			0.98 (0.96,0.99)	**0.007**		
Maximum OC obliqueness angular location, °	0.98 (0.96,0.99)	**0.003**					0.98 (0.96,0.99)	**0.008**
Maximum ALID angular location, °	0.99 (0.97,1)	0.150	0.99 (0.97,1.01)	0.263	0.99 (0.97,1)	0.122	0.99 (0.97,1.01)	0.203

VF: visual field; IOP: intraocular pressure; AL: axial length; CCT: central corneal thickness; MD: mean deviation; ONH: optic nerve head; EOBT: externally oblique border tissue; OC: optic canal; ALID: anterior lamina cribrosa insertion depth

Maximum EOBT angular location, maximum ONH tilt angle angular location, and maximum OC obliqueness angular location were strongly associated with each other; thus, each was analyzed separately in multivariate analysis.

Statistically significant values are shown in bold. ° degree.
